# Hemopexin and HO-1 induction during acute colitis in mice is dependent on interleukin-22

**DOI:** 10.3389/fimmu.2025.1614466

**Published:** 2025-07-28

**Authors:** Ayodeji Samuel Ajayi, Claire Gerkins, Gabriela Fragoso, Annie Calvé, Manuela M. Santos

**Affiliations:** ^1^ Nutrition and Microbiome Laboratory, Institut du Cancer de Montréal, Centre de recherche du Centre hospitalier de l’Université de Montréal (CRCHUM), Montréal, QC, Canada; ^2^ Department of Medicine, Faculty of Medicine, Université de Montréal, Montréal, QC, Canada

**Keywords:** acute colitis, IL-22, hemopexin, heme, bleeding, inflammatory cytokines

## Abstract

**Introduction:**

Inflammatory bowel disease (IBD) is a chronic, relapsing inflammatory disorder of the gastrointestinal tract that frequently requires long-term immunosuppressive therapy, which increases the risk of infections and other complications. During active disease, intestinal bleeding is common and leads to the release of free luminal heme, a pro-inflammatory molecule that can disrupt mucosal integrity, fuel microbial dysbiosis, and amplify inflammation. Interleukin-22 (IL-22) plays a protective role in the gut by promoting epithelial barrier integrity and wound healing. More recently, IL-22 has been shown to induce hemopexin, a heme scavenger protein that limits heme availability and suppresses bacterial growth during systemic infections.

**Methods:**

Here we investigate the protective role of IL-22 and hemopexin in the context of colitis using the dextran sodium sulphate (DSS) acute colitis model in mice. Wild-type (Wt) and *Il22ra1^-/-^* mice were used to evaluate the effects of exogenous hemopexin and hemin treatments on colitis severity.

**Results:**

IL-22 signaling was crucial for the induction of hemopexin in the colon, as *Il22ra1^-/-^* mice exhibited limited hemopexin induction and more severe colitis, which could be reversed by recombinant hemopexin administration. Additionally, hemin treatment, known to upregulate heme oxygenase-1 (HO-1), failed to show full protective effects in *Il22ra1^-/-^* mice, suggesting that IL-22 signaling contributes to the anti-inflammatory and antioxidant effects of hemin by inducing hemopexin and HO-1.

**Discussion:**

These findings reveal a critical protective role for IL-22 by increasing the amount of hemopexin and HO-1 production in the colon, which could be part of a protective mechanism that mitigates DSS-induced colonic inflammation. Given its epithelial-specific and immunomodulatory activity, IL-22 represents a promising therapeutic approach for IBD. Furthermore, hemopexin itself may serve as an adjunct therapy during active disease.

## 1 Introduction

Inflammatory bowel disease (IBD) including Crohn’s disease and ulcerative colitis, is characterized by chronic relapsing intestinal inflammation. Cytokine responses play a crucial role in driving intestinal inflammation in IBD and have been successfully used as targets for therapeutic interventions (1, 2). Interleukin-22 (IL-22) is a member of the IL-10 family that has emerged as an important cytokine in the intestinal environment (3). Its importance is related to its ability to connect immune functions with metabolic functions (4, 5) and with the intestinal environment, as IL-22 levels can be modulated by the gut microbiota (6).

IL-22 is produced in response to inflammatory signals by various innate and adaptive immune cells, primarily innate lymphoid cells (ILCs) and T-helper cells (Th17 and Th22) (7). Unlike many other cytokines that elicit widespread immune activation, IL-22 exerts selective effects on non-hematopoietic cells that express the IL-22 receptor, such as colonic epithelial cells (8). Protective effects of IL-22 in the colon include limiting tissue damage, promoting tissue repair, preventing excessive inflammation (9), and fostering beneficial bacterial communities while limiting the growth of potentially pathogenic or pro-inflammatory organisms (4, 10–12).

Recent studies have revealed the additional protective role of IL-22 during systemic infections through mediation of hemopexin (13), a plasma glycoprotein primarily responsible for binding free heme and mitigating oxidative stress during inflammation (14, 15). Though generally recognized for its systemic effects, hemopexin also has a potential, unexplored role within the colonic microenvironment during acute colitis. This is because the binding of hemopexin to heme both limits heme toxicity to colonic cells and reduces heme availability to microorganisms. Given the role of IL-22 in epithelial protection and tissue homeostasis, along with the growing recognition of the influence of the gut microbiota in IBD (16), we hypothesize that IL-22 signaling may drive hemopexin upregulation as a protective mechanism in colitis.

In this study, we explore the role of IL-22 and IL-22-induced hemopexin in the context of experimental ulcerative colitis using the dextran sodium sulphate (DSS) mouse model of acute colitis.

## 2 Materials and methods

### 2.1 Animal experiments

Animal studies were conducted under the approval of the Institutional Animal Protection Committee (CIPA) at the Centre de recherche du Centre hospitalier de l’Université de Montréal (CRCHUM). *Il22ra1^-/-^* mice and their wild-type (Wt) littermates, of C57BL/6N background, were rederived originally as previously described (Dr Naglaa Shoukry, CRCHUM) (17) and were bred under specific pathogen-free conditions. All mice used for these experiments were females between 8 and 10 weeks old, and they were maintained on a 12-hour light/dark cycle with unlimited access to food (Inotiv Teklad Diets, TD2018, IN, USA) and water.

### 2.2 DSS administration

Acute colitis was induced in mice by subjecting *Il22ra1^-/-^* and Wt to 12 days of 2.5% DSS (DB001; TdB Labs, Upsala, Sweden) treatment in sterile water while the control groups received sterile water. In some experiments, Wt mice received 2.5% DSS in water for 9 days, followed by 3 days of recovery with normal drinking water. The weight of the mice was monitored daily throughout the experiments and the disease activity index (DAI) was evaluated daily by scoring based on stool consistency and rectal bleeding, using the following scale: Stool consistency (Normal =0; Loose =2; Diarrhea =4); Rectal bleeding (Normal =0; Occult blood =2; Rectal bleeding =4) (18, 19). The difference between initial and testing weights was used to determine weight loss, and the presence of persistent watery fecal material in the colon and the lack of fecal pellet development were used to characterize diarrhea. Hemoccult sensa (Beckman Coulter inc, Brea, CA 92821 USA) was used to evaluate bleeding. At day 12, mice were euthanized by intraperitoneal injection of sodium pentobarbital, followed by cervical dislocation. The feces, liver, and colon were collected in 1.5 ml tubes and snap frozen in liquid nitrogen before being transferred to -80°C for further assays.

### 2.3 Treatments

For IL-22 treatment, mice received intraperitoneal injection of recombinant murine IL-22 (rIL-22) (Peprotech, USA) at a dosage of 1 µg/mouse on days 3, 6 and 9 of DSS treatment. Intraperitoneal injections were used to treat mice with hemopexin (Athens Research & Technology, Athens, USA) at a dosage of 5 mg/kg body weight on day 7 of DSS administration, while control mice received an injection of phosphate buffered saline (PBS). For hemin treatment, hemin was dissolved in 0.2 mol/l NaOH and adjusted to pH 7.4 using HCl before being diluted with PBS (Wisent Inc., St-Bruno, QC, Canada). Mice were injected intraperitoneally with 75 µmol/kg of hemin (Sigma-AldrichCo, St Louis, MO, USA) or the vehicle, PBS, on day 5, 8 and 10 of DSS administration.

### 2.4 Heme quantification

The colorimetric method as described by (20) was used with little modification. The assay is based on the chemical conversion of non-fluorescing heme to intensely fluorescent porphyrins (21) and is specific for fecal heme (22). Firstly, colonic contents from mice were promptly snap-frozen and maintained at -80°C until diluted in water 1:1 (w/w). After homogenization, samples were centrifuged for 10 minutes at 1500 × g. 10 µl of the supernatant was then added to 200 μL of glacial acetic acid (ACP Chemicals, Montreal, QC, Canada). Afterwards, 10 μL of fresh aqueous solution of FeSO4.7H_2_O (Sigma-Aldrich, (0.12 mol/l)) and HC1 (Fisherscientific, (4.5 mol/l)) was added. Following an instant 30 minute incubation period at 60°C, 50 μl of the sample was added to 100 μl of a 1:1 2-propanol/water (v/v) mixture. Finally, fluorescence was measured at 360 nm excitation and 594 nm emission.

### 2.5 Protein quantification and ELISA

Fecal and colon samples were first digested by adding 20–30 g to 200 µL radioimmunoprecipitation assay (RIPA) buffer containing NaCl (150 mM), NP-40 (1%), deoxycholic acid (0.50%), SDS (0.10%), Tris pH 8.0 (50 mM) and protease inhibitors (cOmplete^™^, Mini, EDTA free Protease Inhibitor Cocktail Roche) and vortexed for 5 minutes to yield a homogenous suspension. This process is followed by centrifugation (12,000 × g for 10 minutes at 4°C). An aliquot of the supernatant was prepared in a new tube and stored at -20°C until analysis. Protein concentrations were quantified using Pierce™ BCA Protein Assay Kit (Thermo Fisher Scientific, CA, USA). For ELISA, adequate dilutions of homogenates and serum were prepared with PBS containing 0.1% Tween 20 (reagent diluent) or as directed by the manufacturers of the kit. The mouse lipocalin (Lcn)-2/NGAL ELISA kit, (R&D Systems, Minneapolis, MN) and the ELISA Max™ standard set mouse IL-6 and tumor necrosis factor (TNF)-α kits (BioLegend, San Diego, CA, USA; Cerdalane^®^ distributor) were used. Hemopexin concentration was quantified using a mouse hemopexin ELISA kit (Novus Biologicals Bio-Techne, Canada), and heme-oxygenase (HO) using a mouse heme-oxygenase ELISA kit (Abcam inc, Toronto ON, Canada). A multimode microplate reader (Tecan Spark, Morrisville USA) was used to read the absorbance of the plates.

### 2.6 Histology

Sections of the proximal and distal colon were fixed in 10% formalin (ChapTec, Montreal, QC, Canada). The samples were then embedded in paraffin and sectioned at 4 µm-thickness. To assess the severity of colitis, the sections were stained using hematoxylin (RICCA, VWR International, Mississauga, ON) and eosin (H&E; Leica Biosystems Richmond Inc. Richmond, IL).

### 2.7 Quantitative reverse transcriptase-polymerase chain reaction

Total RNA was isolated with Trizol reagent (Invitrogen, Burlington, ON). A second clean-up using the Qiagen mRNA Isolation Kit (Qiagen, Hilden, Germany) was employed to further purify the mRNA in order to prevent DSS from inhibiting downstream reactions. Reverse transcription was performed with Thermoscript RT-PCR System (Invitrogen). Hemopexin (Hpx) mRNA levels were measured by real-time PCR in a Rotor Gene 3000 Real Time DNA Detection System (Montreal Biotech, Kirkland, QC) with PowerUp^TM^ SYBR^TM^ Green Master Mix for qPCR (Thermo Fisher Scientific) as described (23). Expression levels were normalized to the housekeeping gene β-actin. The following primers were used: hemopexin Forward CAGCAGTGGCGCTAAATATCC and hemopexin Reverse ACTCTCCCGTTGGCAGTAGG; β-actin Forward TGTTACCAACTGGGACGACA and β-actin Reverse GGTGTTGAAGGTCTCAAA.

### 2.8 Statistics

Graphpad Prism (Version 10.4.1), Graphpad software, San Diego, CA, USA) was used to analyze all data. Shapiro-Wilk was used for data normality check, while F test (two variances) and Barlett’s test (multiple variances) were used to check for homogeneity of variance. When the data did not pass the Shapiro–Wilk normality test, log(Y) transformation was applied to the data. Statistical significance was determined at *P* values less than 0.05.

## 3 Results

### 3.1 Recombinant IL-22 treatment during the acute phase of colitis induces hemopexin in the colon

**Figure 1:**
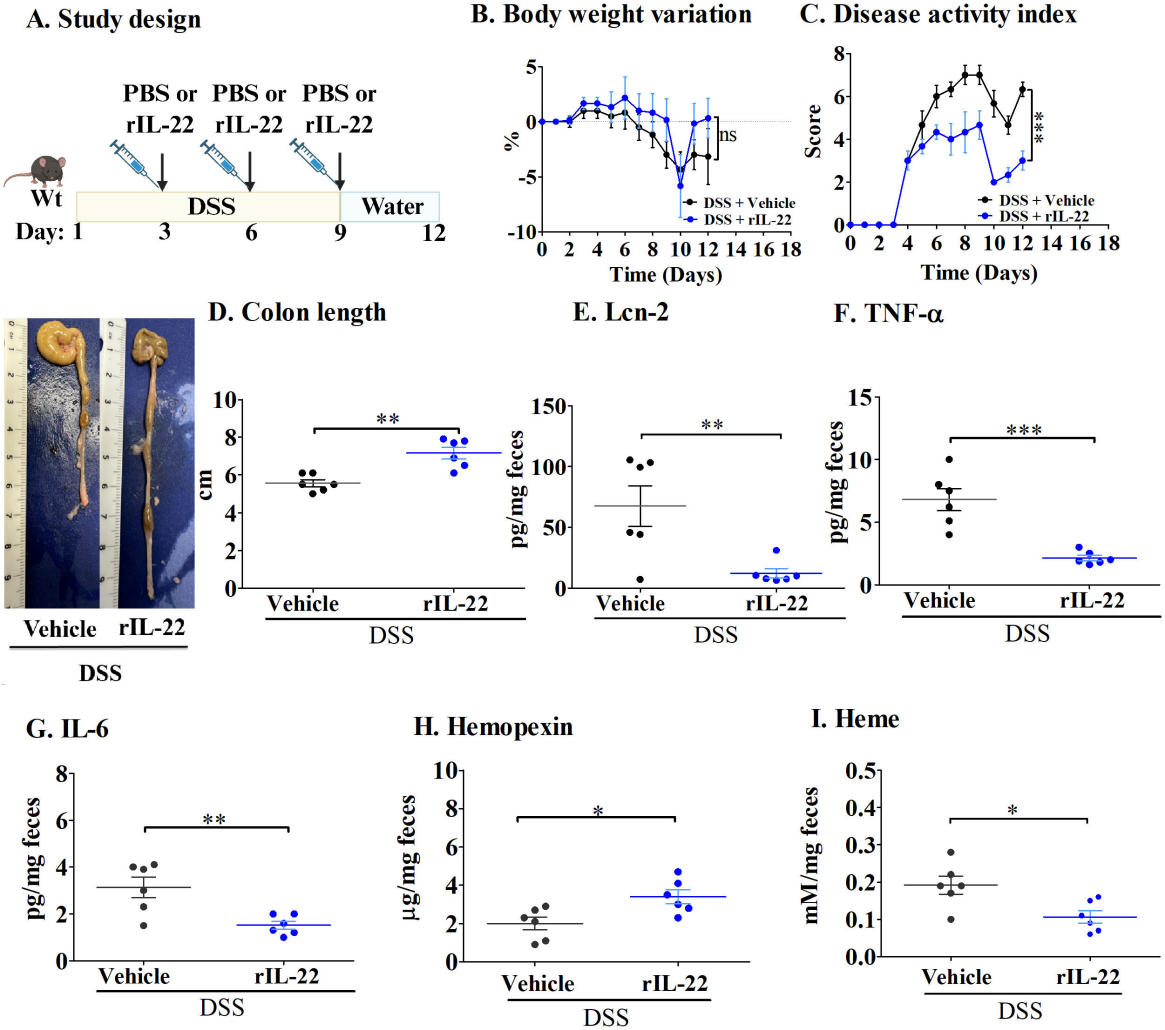
IL-22 treatment lowers inflammation in the DSS-induced mouse model of acute colitis. **(A)** Study design; **(B)** Body weight variation; **(C)** Disease activity index; and **(D)** Colon length. Fecal levels of **(E)** Lcn-2; **(F)** TNF-α; **(G)** IL-6; **(H)** Hemopexin; and **(I)** Heme. Each dot represents one mouse, and means are represented by horizontal bars ± SEM; n=6 mice per group. *P < 0.05, **P< 0.01, ***P < 0.001, ns: not significant. Student’s *t*-test. DSS, dextran sulfate sodium.

Bleeding in the colonic mucosa is commonly seen in both patients and mice with ulcerative colitis, resulting in heme accumulation in the intestine (24). As a defense mechanism against reactive heme, hemopexin is induced (14). To understand the role of IL-22 in hemopexin induction in acute colitis, mice receiving DSS in water were treated with recombinant IL-22 (rIL-22), **Figure 1A**). As shown in **Figure 1B**, no significant differences in body weights were observed between the groups. However, compared with the vehicle-treated control mice, rIL-22 significantly decreased DAI scores during DSS treatment (**Figure 1C**).

During DSS treatment, the epithelium becomes injured, causing increased cell death, thereby leading to the shortening of the colon (18). rIL-22 treatment significantly reduced this effect, with these mice presenting longer colons than PBS-treated mice (**Figure 1D**). In addition, mice treated with rIL-22 had decreased levels of inflammatory cytokines, namely Lcn-2 (**Figure 1E**), one of the most sensitive markers of inflammation in IBD patients (25), as well as TNF-α and IL-6 (**Figures 1F, G**). Most importantly, rIL-22 treatment enhanced hemopexin levels in fecal samples, resulting in reduced heme levels (**Figures 1H, I**).

These results indicate that hemopexin is induced by IL-22, and that it has a protective effect in acute colitis, limiting DSS-induced damage of colonic epithelial cells and lessening inflammation.

### 3.2 The absence of IL-22ra1 signaling blunts hemopexin induction and aggravates acute colitis in mice

**Figure 2:**
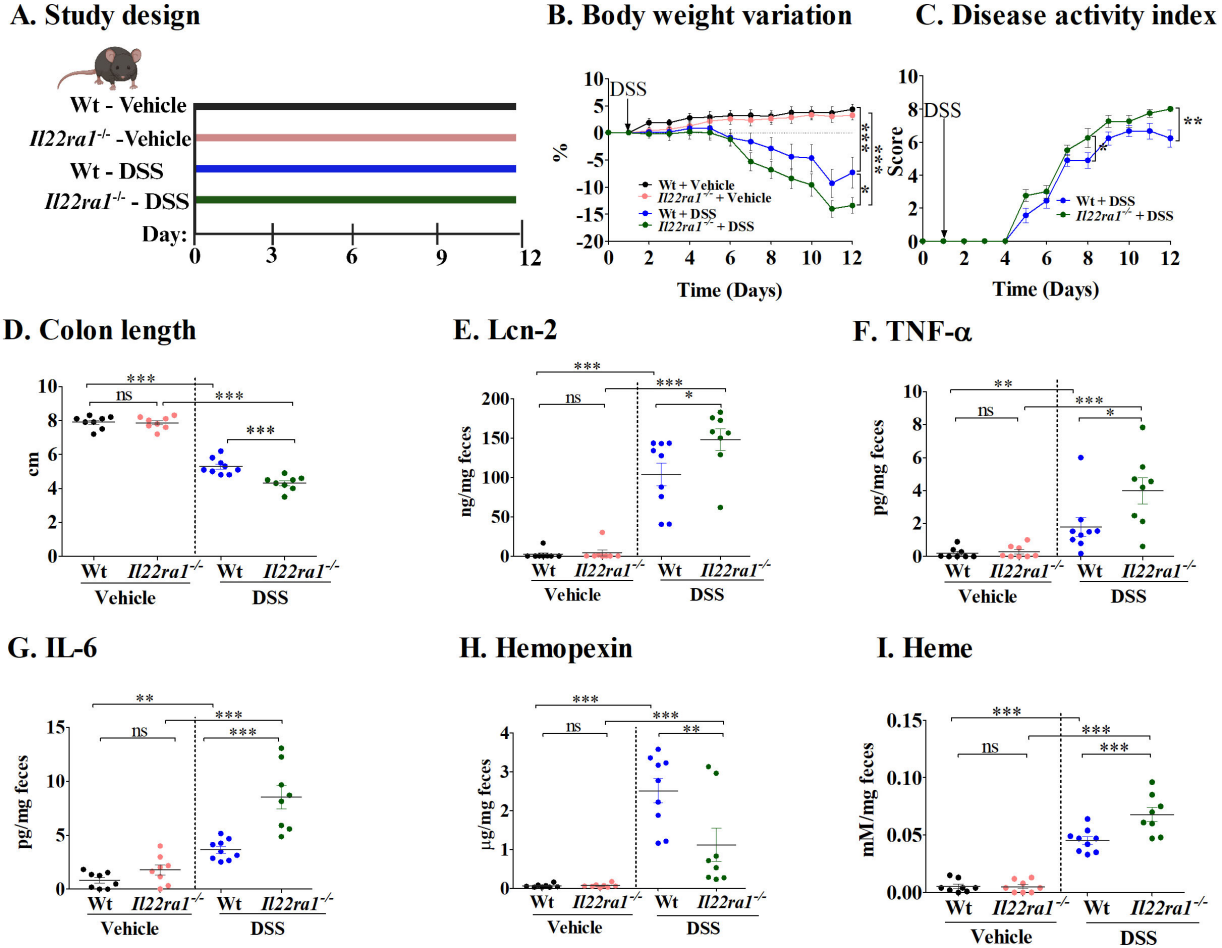
Increased colitis severity in DSS-treated IL-22ra1 knockout mice. **(A)** Study design; **(B)** Body weight variation; **(C)** Disease activity index; **(D)** Colon length; Fecal levels of **(E)** Lcn-2; **(F)** TNF-α; **(G)** IL-6; **(H)** Hemopexin; and **(I)** Heme. Each dot represents one mouse, and means are represented by horizontal bars ± SEM; n=8–9 mice per group. ANOVA *P < 0.05, **P < 0.01,***P < 0.001, ns: not significant). DSS, dextran sulfate sodium.

To further understand the role of hemopexin induced through IL-22 signaling, we next performed experiments in *Il22ra1^-/-^* mice (**Figure 2A**). Following DSS treatment, *Il22ra1^-/-^* mice showed significantly more weight loss (**Figure 2B**) and higher DAI scores at the endpoint than Wt mice (**Figure 2C**). In addition, DSS-treated *Il22ra1^-/-^* mice had significantly shorter colon lengths compared to the Wt mice (**Figure 2D**). Furthermore, DSS-treated *Il22ra1^-/-^* mice showed significantly higher levels of fecal Lcn-2, TNF-α, and IL-6 (**Figures 2E–G**). Conversely, compared to the DSS-treated Wt mice, DSS-treated *Il22ra1^-/-^* mice had a significantly reduced ability to induce hemopexin mRNA in the liver, thus resulting in lower hemopexin levels in the serum (**Supplementary Figures S1A, B**) and, critically, in feces (**Figure 2H**). The reduction of hemopexin in *Il22ra1^-/-^* mice was accompanied by an increase in fecal heme levels (**Figure 2I**).

Taken together, these results underscore the importance of IL-22ra1 signaling in mediating systemic and colonic hemopexin levels and reducing colitis severity in response to DSS.

### 3.3 Hemopexin administration reverses colitis aggravation in the absence of IL-22ra1 signaling

**Figure 3:**
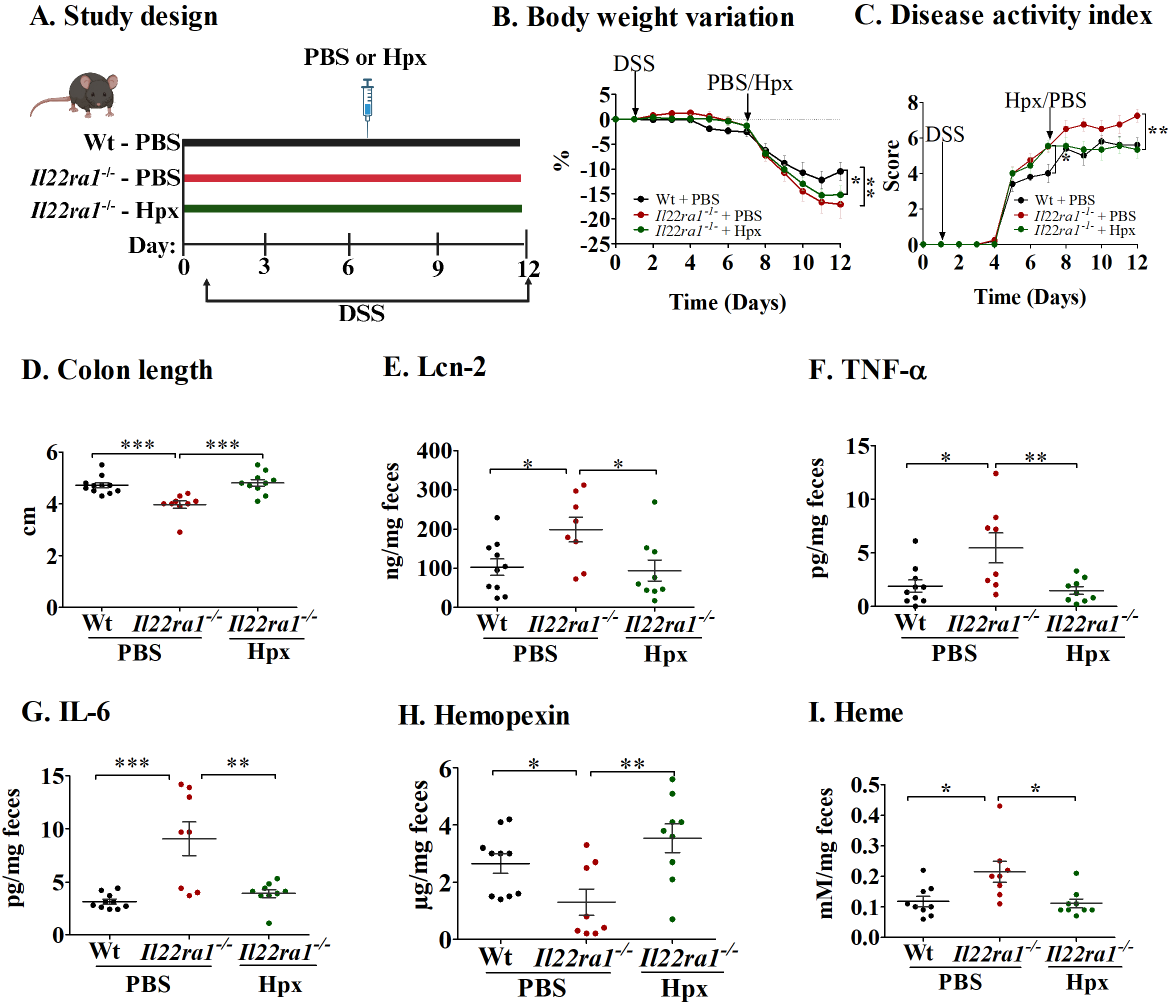
Hemopexin treatment decreases inflammation in the absence of IL-22ra1 signaling in mice. **(A)** Study design; **(B)** Body weight variation **(C)** Disease activity index; **(D)** Colon length. Fecal levels of **(E)** Lcn-2; **(F)** TNF-α; **(G)** IL-6; **(H)** Hemopexin; and **(I)** Heme. Each dot represents one mouse, and means are represented by horizontal bars ± SEM; n=8–10 mice per group. ANOVA, *P < 0.05, **P < 0.01, ***P < 0.001, ns: not significant. DSS, dextran sulfate sodium; PBS, phosphate-buffered saline; Hpx, hemopexin.

To further evaluate the importance of IL-22-dependent hemopexin induction on the severity of DSS-induced acute colitis, we treated *Il22ra1^-/-^* mice with exogenous hemopexin (**Figure 3A**). Despite similar body weights between PBS-treated (control) and hemopexin-treated *Il22ra1^-/-^* mice during the experiment (**Figure 3B**), colitis severity was significantly attenuated by exogenous hemopexin treatment as indicated by lower DAI scores (**Figure 3C**) and improved colonic lengths when compared to PBS treatment (**Figure 3D**). DSS-treatment caused visible changes in pathological parameters including crypt distortion, epithelial damage, ulceration, and inflammatory cell infiltration. The overall histological damage was more severe in *Il22ra1^-/-^* compared to Wt mice, and was attenuated by hemopexin treatment (**Supplementary Figure S2**). Consistently, hemopexin administration significantly reduced fecal Lcn-2, TNF-α, and IL-6 levels in hemopexin-treated *Il22ra1^-/-^* mice, which showed similar levels to DSS-treated Wt mice (**Figures 3E–G**). Finally, levels of fecal hemopexin were increased after exogenous hemopexin treatment, and concordantly, fecal heme levels significantly decreased (**Figures 3H, I**).

Together, these results show that exogenous hemopexin has a protective effect in acute colitis and further indicate that hemopexin induction during colitis is dependent on IL-22ra1 signaling.

### 3.4 Full anti-inflammatory and anti-oxidant effects of hemin require functional IL-22ra1 signaling

**Figure 4:**
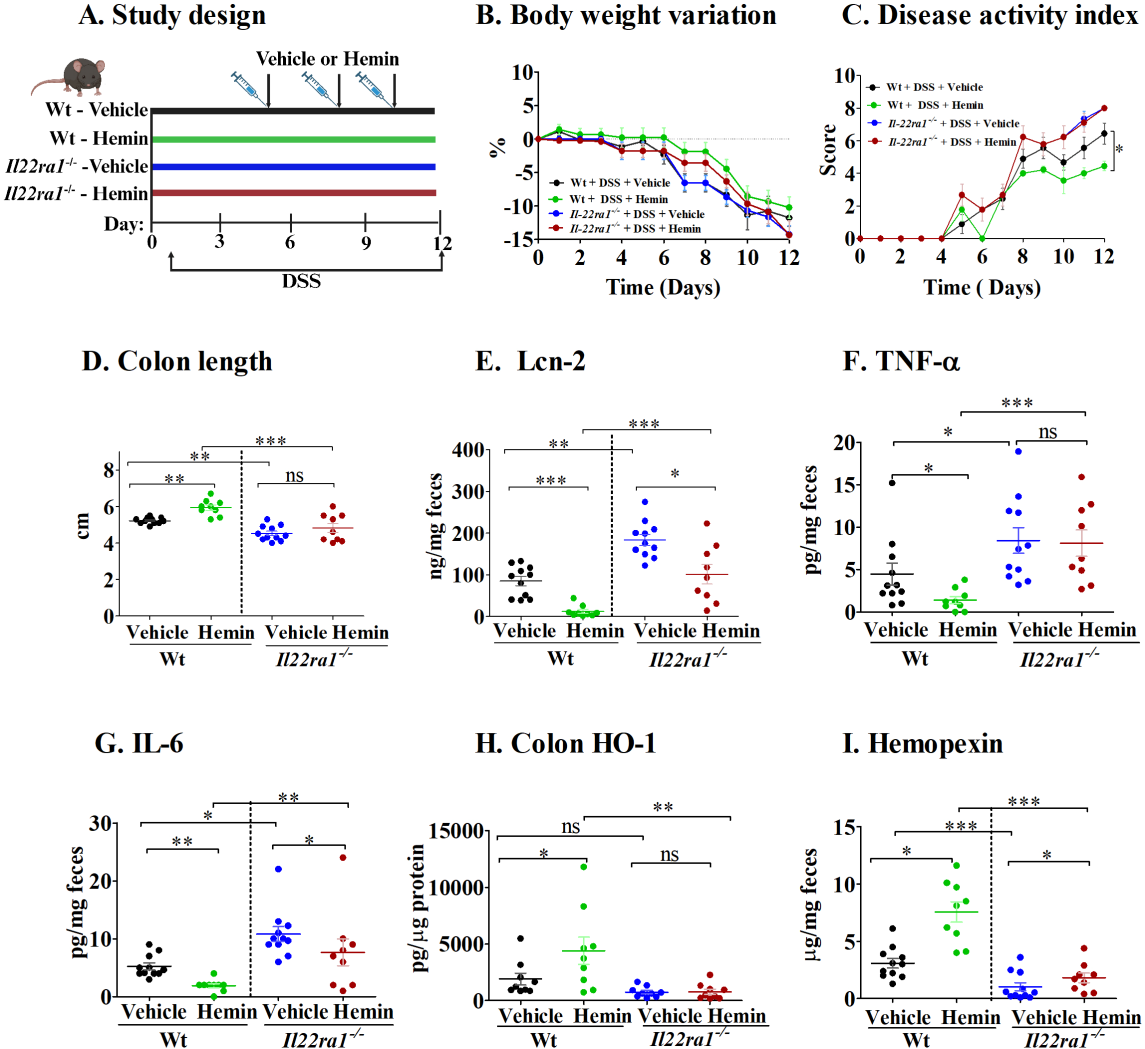
The effect of hemin treatment is attenuated in *Il22ra1^-/-^* mice. **(A)** Study design; **(B)** Body-weight variation; **(C)** Disease activity index; **(D)** Colon length. Fecal levels of **(E)** Lcn-2; **(F)** TNF-α; **(G)** IL-6; **(H)** Colon HO-1. **(I)** Fecal hemopexin. Each dot represents one mouse, and means are represented by horizontal bars ± SEM; n=9-11 mice per group. ANOVA, *P < 0.05, **P < 0.01,***P < 0.001). ns, not significant between the groups; DSS, dextran sulfate sodium.

Next, we investigated the role of IL-22 in hemopexin induction (14) by treating mice with hemin, the ferric form of heme with a chloride ligand, during experimental acute colitis (**Figure 4A**). Hemin is the substrate and primary inducer of heme-oxygenase (HO-1), the rate limiting enzyme that catalyzes the breakdown of heme to carbon monoxide (CO), biliverdin and free iron (26, 27). As such, hemin has both anti-inflammatory and anti-oxidant properties (28, 29). As seen in **Figure 4B**, body weights remained similar among all mouse groups. However, colitis severity was significantly reduced in Wt mice that received hemin compared to Wt mice that received the vehicle, as indicated by reduced DAI scores and increased colon lengths (**Figures 4C, D**). In marked contrast, hemin treatment in *Il22ra1^-/-^* mice failed to attenuate colitis severity (**Figures 4C, D**). In addition, while hemin caused a significant reduction in fecal Lcn-2 and IL-6 levels in both Wt and *Il22ra1^-/-^* mice (**Figures 4E–G**), levels in hemin-treated *Il22ra1^-/-^* mice were significantly higher than those in hemin-treated Wt mice. Consistent with these effects, HO-1 production was significantly enhanced in the colon of Wt mice, but not in *IL22ra1* ^-/-^ mice (**Figure 4H**). While hemopexin levels were enhanced in both the Wt and *Il22ra1^-/-^* mice that received hemin, this elevation was much more modest in *Il22ra1^-/-^* mice (**Figure 4I**).

These results suggest that the attenuation of acute colitis by hemin treatment depends on intact IL-22ra1 signaling, involving hemopexin induction and downstream antioxidant effects through HO-1 production.

## 4 Discussion

The protective role of IL-22 in IBD is due to the promotion of epithelial barrier integrity, enhancement of tissue repair, and regulation of immune responses. Here we report that IL-22 is additionally protective against heme toxicity through the modulation of colonic hemopexin and HO-1 levels in the DSS-induced mouse model of acute colitis.

We show that recombinant IL-22 administration significantly alleviated inflammation in DSS-treated mice while conversely, IL-22ra1 deficiency exacerbated colitis severity in DSS-treated mice. These findings are in line with previous reports that IL-22 deficiency results in exacerbated DSS-mediated colitis due to alterations to the colonic microbiota (4) and compromised colonic epithelial integrity during gastrointestinal infections (30). Most importantly, we provide evidence that, in addition to the reported role of IL-22 in promoting epithelial regeneration and wound healing (31), IL-22 is also essential for the appropriate modulation of hemopexin levels in the colon. This was evidenced by the limited hemopexin induction in the liver and its reduced presence in the serum and feces of *Il-22ra1* ^-/-^ mice and by the consequent severity of DSS-induced colitis, which could be reversed by recombinant hemopexin treatment. In addition, the protective effects of hemin treatment, including the upregulation of HO-1 (32), were significantly reduced in *Il-22ra1* ^-/-^ mice. HO-1 has been shown to play a protective role in DSS-induced intestinal inflammation (33), with previous research showing that IL-22 can directly stimulate HO-1 in keratinocytes (34) and in the liver (35). In this study, we demonstrate that HO-1 is additionally induced in an IL-22-dependent manner in the colon. HO-1 expression can be triggered by various stimuli in gut epithelial cells (36) as well as in gut resident macrophages (37). More precisely, administration of cobalt protoporphyrin IX was shown to activate HO-1 in gut macrophages in the azoxymethane/DSS mouse model of tumorigenesis, thus decreasing tumor burden (37). Likewise, in a DSS model of acute colitis, HO-1 activation by cobalt protoporphyrin limited colonic inflammation (38). Furthermore, in peritoneal macrophages, the heme-hemopexin complex has been shown to activate HO-1 (39). Therefore, in addition to the direct effect of IL-22 in inducing HO-1, IL-22 signaling may also contribute to the anti-inflammatory and anti-oxidant effects of hemin (40) by activating the heme-hemopexin-HO-1 axis.

In the context of IBD, the presence of heme in the luminal space of the colon due to either the diet (red meat consumption) or intestinal bleeding has a direct cytotoxic effect on the colonic epithelium (41), further aggravating acute colitis (42). Heme injures the colon surface epithelium by generating cytotoxic and oxidative stress resulting in mucosal hyperproliferation (43). In addition, luminal heme levels may further exacerbate colitis indirectly through the modulation of the gut microbiota composition and function (44).

Cytokines are central to the pathology of IBD, making them attractive therapeutic targets (45). A key advantage of targeting IL-22 in IBD is that IL-22 receptors are primarily expressed on epithelial cells rather than immune cells, meaning that IL-22-based therapies can enhance tissue repair without directly suppressing systemic immune responses. This selective action reduces the risk of systemic immunosuppression-associated complications, a major concern with many current IBD treatments that broadly target inflammation (46), such as anti-TNF-α therapy (47).

**Figure 5:**
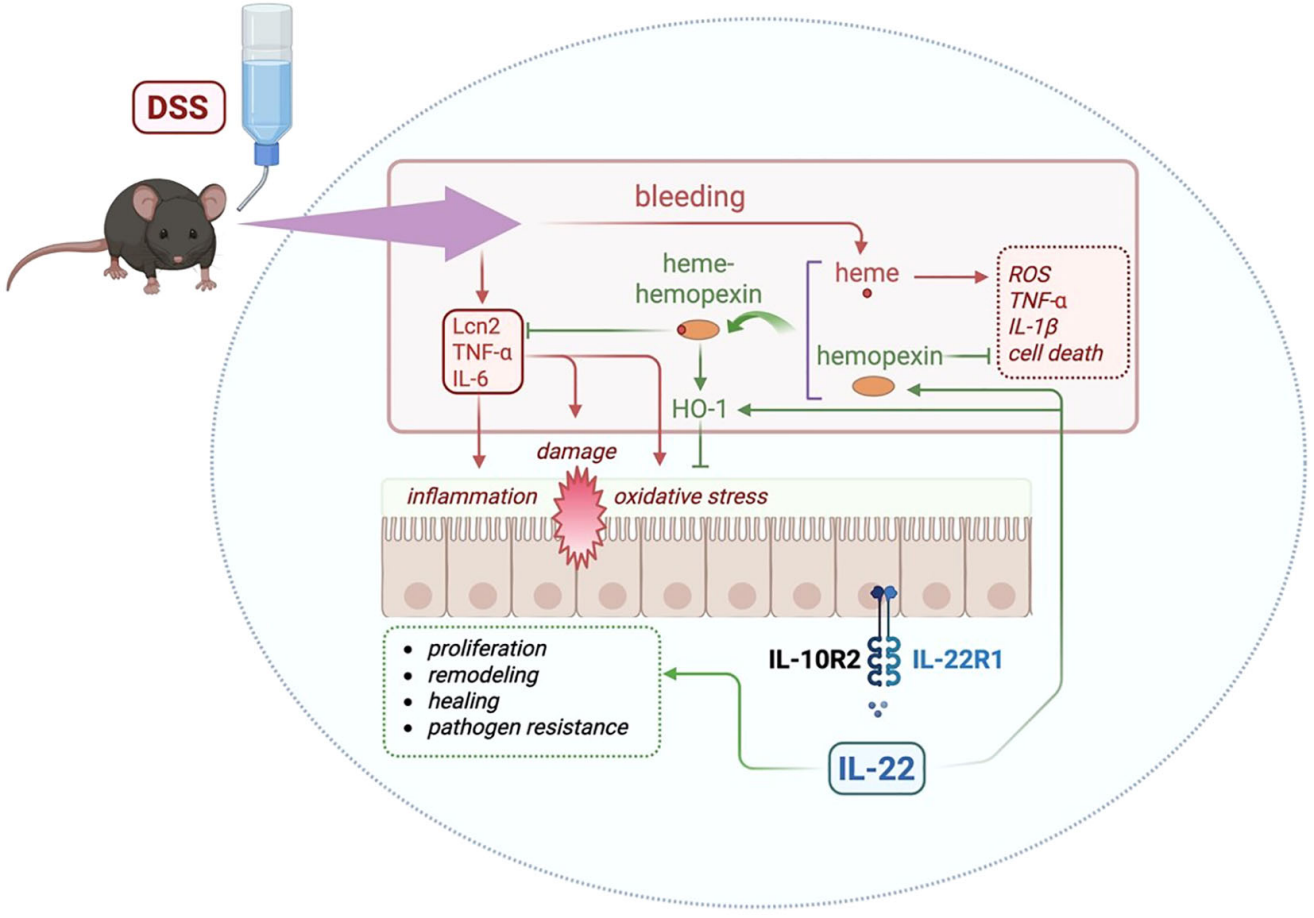
Graphical abstract summarizing the major findings of this study and the proposed mechanism. DSS administration triggers epithelial damage, bleeding, and the release of free heme. IL-22 enhances the levels of hemopexin, a heme-binding protein that sequesters free heme, reducing inflammation and tissue damage. The heme-hemopexin complex facilitates detoxification of heme and promotes the induction of HO-1, an antioxidant enzyme, reducing oxidative stress. In addition, IL-22 can directly induce HO-1. Previously described roles for IL-22 in the gut includes cell proliferation, tissue remodeling, wound healing, and antimicrobial defense (dashed green box). Previous reports show that hemopexin can inhibit reactive oxygen species (ROS), inflammatory cytokines (TNF-α and IL-1β), and cell death (dashed red box), which can be induced by free heme (48). Blunt arrows (┴) indicate inhibition while sharp arrows (→) indicate stimulation. Created with Biorender.com.

In conclusion, our findings highlight the important role of IL-22-dependent hemopexin and HO-1 induction in the context of acute colitis in mice (**Figure 5**). Our current study design does not allow for a clear separation of the individual contributions of HO-1 and hemopexin in the IL-22-mediated protective response. Future research using, for example, hemopexin knockout mice (49) and HO-1 inhibitors (50) should examine further the distinct roles of IL-22-induced hemopexin and IL-22-induced HO-1. In addition to its well-known role in heme scavenging and oxidative stress reduction, hemopexin may also influence gut microbiota dynamics by limiting heme availability to potential pathobionts and pathogens. Given the increasing recognition of microbial dysbiosis in IBD pathogenesis, targeting heme metabolism through hemopexin offers an intriguing therapeutic strategy. The integration of IL-22 and hemopexin-based therapies could represent a novel and complementary therapeutic avenue for IBD treatment, balancing tissue repair with controlled inflammation.

## Data Availability

The raw data supporting the conclusions of this article will be made available by the authors, without undue reservation.

## Supplementary material

Supplementary Figure 1
**(A)** Liver hemopexin mRNA levels. **(B)**; Serum hemopexin levels. Each dot represents one mouse, and means are represented by horizontal bars ± SEM; n=8–10 mice per group. ANOVA, **P* < 0.05 and ****P* < 0.001, ns: not significant. DSS = dextran sulfate sodium, Hpx, hemopexin.

Supplementary Figure 2 Histological assessment of DSS-induced colitis. Representative hematoxylin and eosin (H&E)-stained colon sections. Magnification: 200x, DSS, dextran sulfate sodium; PBS, phosphate-buffered saline; Hpx, hemopexin.
