# Supplementary figures and images for: Hemopexin and HO-1 induction during acute colitis in mice is dependent on interleukin-22
Source: Front Immunol. 2025 Jul 28;16:1614466. doi: 10.3389/fimmu.2025.1614466 (PMC12336042; doi:10.3389/fimmu.2025.1614466)

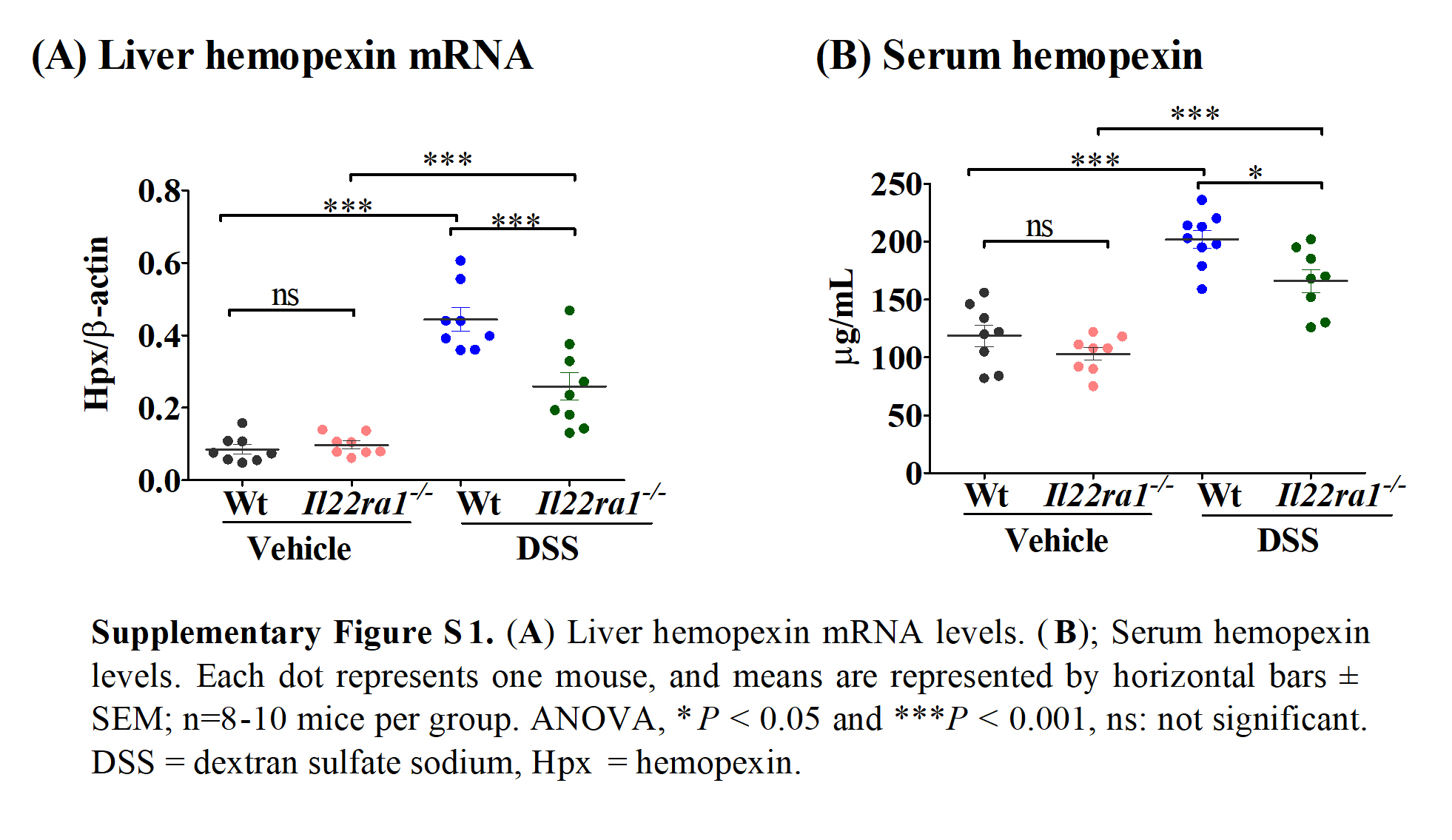

Supplement: Supplementary Figure 1 — (A) Liver hemopexin mRNA levels. (B); Serum hemopexin levels. Each dot represents one mouse, and means are represented by horizontal bars ± SEM; n=8–10 mice per group. ANOVA, *P < 0.05 and ***P < 0.001, ns: not significant. DSS = dextran sulfate sodium, Hpx, hemopexin. [file Image1.jpg]

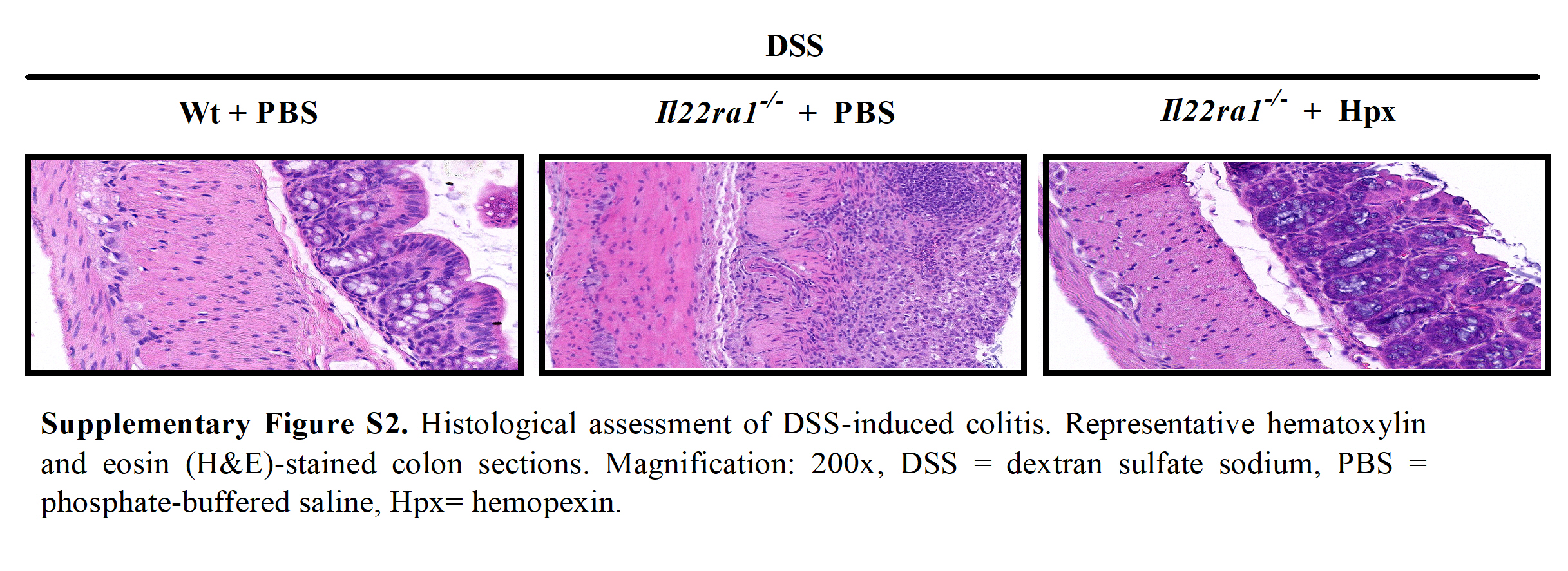

Supplement: Supplementary Figure 2 — Histological assessment of DSS-induced colitis. Representative hematoxylin and eosin (H&E)-stained colon sections. Magnification: 200x, DSS, dextran sulfate sodium; PBS, phosphate-buffered saline; Hpx, hemopexin. [file Image2.jpg]
